# Comparison meta-analysis of intraoperative MRI-guided needle biopsy versus conventional stereotactic needle biopsies

**DOI:** 10.1093/noajnl/vdad129

**Published:** 2023-10-10

**Authors:** Sanjay Dhawan, Clark C Chen

**Affiliations:** Department of Neurosurgery, University of Minnesota, Minneapolis, Minnesota, USA; Department of Neurosurgery, University of Minnesota, Minneapolis, Minnesota, USA

**Keywords:** brain tumor, intraoperative MRI, meta-analysis, needle biopsy, stereotactic

## Abstract

**Background:**

MRI-guided needle biopsy (INB) is an emerging alternative to conventional frame-based or frameless stereotactic needle biopsy (SNB). Studies of INB have been limited to select case series, and comparative studies between INB and SNB remain a missing gap in the literature. We performed a meta-analysis to compare INB and SNB literature in terms of diagnostic yield, surgical morbidity and mortality, tumor size, and procedural time.

**Methods:**

We identified 36 separate cohorts in 26 studies of SNB (including both frameless and frame-based biopsies, 3374 patients) and 27 studies of INB (977 patients). Meta-regression and meta-analysis by proportions were performed.

**Results:**

Relative to publications that studied SNB, publications studying INB more likely involved brain tumors located in the eloquent cerebrum (79.4% versus 62.6%, *P* = 0.004) or are smaller in maximal diameter (2.7 cm in INB group versus 3.6 cm in the SNB group, *P* = .032). Despite these differences, the pooled estimate of diagnostic yield for INB was higher than SNB (95.4% versus 92.3%, *P* = .026). The pooled estimate of surgical morbidity was higher in the SNB group (12.0%) relative to the INB group (6.1%) (*P* = .004). Mortality after the procedure was comparable between INB and SNB (1.7% versus 2.3%, *P* = .288). Procedural time was statistically comparable at 90.3 min (INB) and 103.7 min (SNB), respectively (*P* = .526).

**Conclusions:**

Our meta-analysis indicates that, relative to SNB, INB is more often performed for the challenging, smaller-sized brain tumors located in the eloquent cerebrum. INB is associated with lower surgical morbidity and improved diagnostic yield.

**Key Points**

- MRI-guided needle biopsy (INB) might be associated with more favorable diagnostic yield and surgical morbidity over conventional stereotactic needle biopsy.
- Lesions biopsied with intraoperative MRI guidance are more likely to be smaller in size.
- INB more likely involved brain tumors located in the eloquent cerebrum.

Molecular and histologic characterization of surgically acquired tumor tissue remains the foundation of modern neuro-oncology,^1^ and stereotactic needle biopsy (SNB) is the mainstay surgical technique for achieving such diagnosis when risks outweigh the benefits of an open surgical procedure.^2^ The diagnostic yield for SNB is generally favorable, ranging 84–100%.^3,4^ However, the likelihood of definitive tissue diagnosis decreases with smaller tumors or tumors with significant regional histologic heterogeneity.^5^ In cases where tissue diagnosis is not achieved in the first procedure, a second SNB achieved definitive diagnosis in 90% of the cases,^6^ suggesting technical challenges associated with SNB rather than the intrinsic properties of the lesion as the underlying cause for diagnostic failures. While the likelihood of procedural complication is low, with reported postoperative morbidity generally <10%,^7,8^ the likelihood of complication increases with the number of samples taken,^9^ with the number of trajectories to the lesion,^10^ and for lesions in the eloquent cerebrum or deep gray matter/brainstem.^10^ Though most procedural complications are tolerated or self-resolving, devastating sequelae, including mortality, do occur in 1–4% of patients after SNB.^7,11^

Conventional SNB is performed using frame-based or frameless stereotaxy. The diagnostic yield and safety profile of both techniques is comparable for tumors > 1 cm in maximal diameter.^12,13^ For both procedures, target localization is based on triangulation of the target relative to fiducials of known spatial coordinate,^14^ without direct visualization of the target during the procedure. While the mathematics underlying this methodology is elegant,^15^ the accuracy of the procedure can be affected by several elements, including technical errors,^16^ unintended movements of the fiducials,^16,17^ CSF egression during the procedure,^18–20^ and poor equipment maintenance/calibration.^18^ Unfortunately, real-time visual confirmation that the actual biopsy site coincides with the intended target site is not possible with conventional SNB.^21^ In cases where the frozen pathology of the SNB-acquired samples revealed nondiagnostic tissue, the surgeon is left in the precarious position of making surgical adjustments without understanding the root cause.

The technological convergence of intraoperative magnetic resonance imaging (iMRI) and MRI-compatible stereotactic systems has led to the development of Intraoperative MRI-guided needle biopsy (INB). Key benefits of INB involve the near-real-time visualization of the actual trajectory relative to the planned trajectory as well as opportunities for intraoperative trajectory adjustment.^21^ For instance, if the location of the Burr hole is suboptimal to support the trajectory, the Burr hole and/or the planned trajectory can be modified in real-time.^22^ Similarly, if the lesion has shifted as a result of pneumocephalus or CSF egression, the planned trajectory can be modified accordingly.^20,22^ Moreover, imaging between biopsies affords assessment of biopsy-related complications such as hematoma formation and allows the surgeon to adjust surgical decisions accordingly.^23^

There has been a growing literature describing the effectiveness and safety profiles of INBs.^9,13^ Here, we reviewed the available INB literature and performed a comparative meta-analysis of this literature relative to our previously published results for SNB.

## Methods

### Search Algorithm

This systematic review and meta-analysis were conducted in accordance with the PRISMA guideline.^24^ A comprehensive PubMed database search for articles focusing on intraoperative MRI guidance for needle biopsies primarily for brain tumor indications was performed.^9^

The following search strategy was used: ((stereotactic* OR stereotaxis* OR frame based* OR frameless*)[Title/Abstract] AND (biopsy* OR neuro biopsy* OR resection* OR surgery*)[Title/Abstract] AND ((brain* OR neurosurgery* OR neurosurgeon* OR intracranial* OR neurological* OR neuropathology* OR cerebral*OR central nervous system*)[Title/Abstract] OR brain/pathology[MeSH Terms] OR brain diseases[MeSH Terms])) NOT case reports.^9,13^ The protocol for this meta-analysis titled “Supplemental tools assisting intracranial stereotactic biopsy” was registered with PROSPERO (#CRD42020152735) on 28 April, 2020.^25^

The studies included in the meta-analysis followed the following inclusion criteria: (1) written in English (or English language translation available); (2) involved human subjects; (3) fully reported peer-reviewed clinical studies; (4) studies reporting diagnostic yield, morbidity, mortality, operative time, and site of lesion biopsied with intraoperative MRI guidance for needle biopsies.

Two authors independently extracted the following data from the included articles (S.D. and C.C.C.). Pertinent data for the size of the lesion was also extracted from the studies. For studies reporting the volume of the lesions, *V* (cm^3^, or cc) or area of lesions, *A* (cm^2^), approximate lesion size was calculated using the formula: *r* = 2*x* ∛ *V*.(3/4π) or *r* = √*A*/ π, respectively. Data for frame-based and frameless needle biopsy groups was used from our previous publications.^9,13^ Overall, the variables used in data compilation and analysis were: first author, year of publication, *Q* score (Newcastle–Ottawa quality assessment score),^26^ methods of needle biopsy (FB/FL, intraoperative MRI), diagnostic yield, morbidity, mortality, operative time, and size of the biopsied lesion.

Quality (*Q*) score was assigned to each study using the Newcastle–Ottawa scale in a similar manner as our previously published analyses.^9,13,26^ The studies included in this analysis were assessed in a similar manner. A score of 1(versus 0) was assigned for satisfactory fulfillment of each criterion. Studies with a NOS ≥ 5 were classified as high-quality studies and those with NOS < 5 were categorized as low-quality studies.^9,13^

### Statistical Analysis

DerSimonian and Laird random effects model was applied to pool the meta-analysis results.^27^ Meta-analysis by proportions and meta-regression analyses were performed. The event rates for diagnostic yield, morbidity, and mortality were calculated using meta-analysis by proportions. The effect size was reported in terms of odds ratio (OR) for diagnostic yield, morbidity, and mortality, and in standard difference in means (SDM) for surgical time and maximum tumor diameter, with a 95% confidence interval. Meta-regression analysis was used to compare event rates for DY, morbidity and mortality; SDM for surgical time, and mean size of the lesion between the INB and SNB groups. Subgroup analysis was used to compare the proportion of patients across INB and SNB groups who underwent biopsy in the eloquent area of the cerebrum. Eloquent areas included left frontal, left temporal, bilateral parietal and occipital lobes, corpus callosum, pineal region, and deep gray matter-basal ganglia, thalamus and brainstem.

Similar to our previous analysis, heterogeneity across the studies was gauged using the Higgins inconsistency index (*I*^2^) and Cochran’s *Q* χ² test.^28^ High, moderate, and absence of heterogeneity was reported if *I*^2^ was >50%, 25–50% or <25%, respectively.^28^

Funnel plots, Egger’s regression intercept test, and Duval and Tweedie’s trim and fill test were used to assess the publication bias.^29,30^ The overall stability of our analysis was determined using a cumulative meta-analysis, performed after arranging the studies from largest to smallest w.r.t sample size (and from most to least precise) in a similar fashion as performed in our previous publications.^9,13^ The influence of individual studies was ruled out by performing sensitivity analysis by excluding one study at a time.^31^

Comprehensive meta-analysis (CMA) software, version 3.3070 Biostat, Englewood, New Jersey, USA was used for our analysis. *P*-value < 0.05 was considered statistically significant.

## Results

### Study Selection

**Figure 1.**
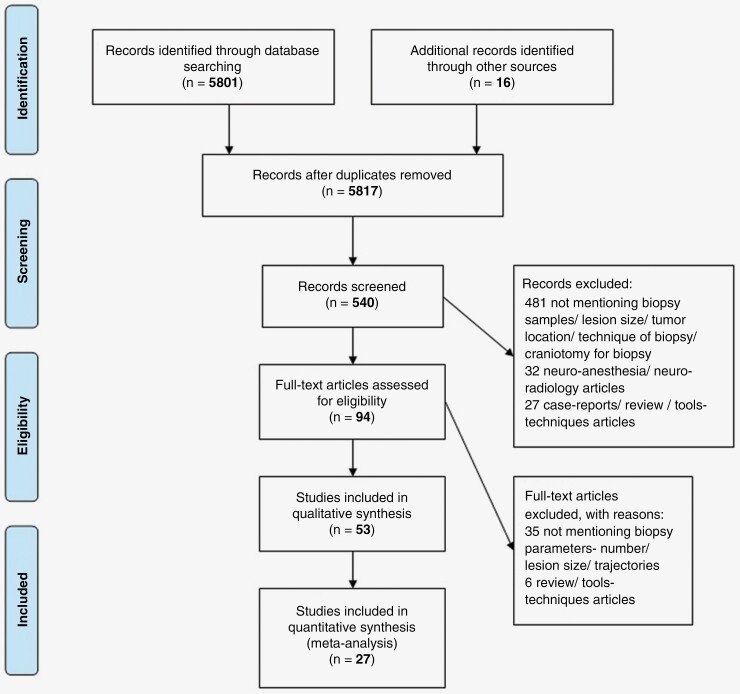
Preferred reporting items for systematic reviews and meta-analysis (PRISMA) flow diagram for article search and study selection.

Out of the total 5801 studies that were identified from the PubMed database in our previous study,^9,13^ 540 pertinent studies focused on supplemental tools for needle biopsy. Intraoperative MRI was selected for manual screening by title and abstract. Sixteen studies added from references mentioned in other studies were additionally screened. Ninety-four studies were reviewed as full text after excluding 430 studies not directly related to intraoperative MRI or frame-based/frameless needle biopsies. Fifty-three out of 94 studies focused on intraoperative MRI guidance for needle biopsies. After further screening, 27 studies were included in the meta-analysis (Figure 1).

### Study Characteristics

Twenty-seven studies included in our meta-analysis were published from 1999 to 2023 and were either prospective observational studies or retrospective analyses.^4,21,22,32–55^ Demographics of individual studies are shown in Table 1.

**Table 1.** Demographics of studies included in the INB group. *Q*: Newcastle–Ottawa Score

Study	Year	*Q* score	Sample Size (*n*)	Age (y)	Male Gender, *n* (%)
Taha^22^	2022	5	52	–	–
Sterk^32^	2022	6	10	56.3	7 (70)
Bartek^33^	2019	6	1	19	1 (100)
He^34^	2019	6	16	52	–
Akay^35^	2019	4	16	–	–
Lu^36^	2019	6	50	45.3	30 (60)
Carroll^37^	2018	5	1	38	0
Zhang^38^	2017	6	39	44.8	21 (53.8)
Scheer^4^	2017	6	11	55.7	6 (54.5)
Yao^39^	2016	6	24	49.7	15 (62.5)
Lu^21^	2015	5	112	–	–
Burkhardt^40^	2013	6	78	57.5	46 (58.9)
Czyz^41^	2013	6	21	51.7	14 (66.6)
Czyz^42^	2012	6	15	52	9 (60)
Schulder^43^	2011	6	39	49	25 (64.1)
Quinn^44^	2010	6	33	44	21 (63.6)
Nimsky^45^	2004	6	19	50.7	9 (47.3)
Bernays^46^	2002	6	114	53	–
Kanner^47^	2002	4	15	–	–
Hall^48^	2001	5	38	47	28 (73.6)
Fontaine^49^	2000	6	100	52	57 (57)
Bernstein^50^	2000	6	10	55.6	5 (50)
Moriarty^51^	2000	4	68	–	–
Staubert^52^	2000	4	26	–	–
Rubino^53^	1999	6	13	56	11 (84.6)
Hall^54^	1999	5	35	48	22 (62.8)
Kollias^55^	1998	5	21	50.7	10 (47.6)

Using the Newcastle–Ottawa scale, 23 studies were categorized as high-quality, and 4 were graded as low-quality studies. In total, the 26 studies yielded results for 3374 patients in the FB/FL group, and 27 studies yielded results for 977 patients in the INB group. The clinical characteristics of each study in the INB group are shown in Table 2. The distributions of these clinical variables are comparable to those previously published for SNB.^13^ The mean age of patients in the study cohorts ranged from 19 to 57.5 years. The predominant pathology was glioblastoma. The main difference between the INB and SNB studies were: (1) the mean maximum diameter of INB biopsied lesions ranged from 0.8 to 5.3 cm and SNB biopsied lesions 3.1 to 5.1 cm in FB/FL group (Table 2), (2) 79.4% INB biopsied patients harbored lesions in eloquent cortex whereas 62.6% in the SNB group harbored lesions in the eloquent cortex (Table 2).

**Table 2.** Clinical characteristics of studies included in the INB group.^*^The median size of the lesion. ^¶^Distribution of tumor location not clearly mentioned

Study	Diagnostic Yield, *n* (%)	Morbidity, *n* (%)	Mortality, *n* (%)	Mean Lesion Size (cm)	Eloquence (n,%)	Procedural Time (min)
Taha^22^	52 (100)	0 (0)	0 (0)	1.7 ± 0.8	Yes (33, 63.4)	138.5 ± 4.8
Sterk^32^	10 (100)	0 (0)	0 (0)	0.9 ± 0.2	Yes (10, 100)	114 ± 42.8
Bartek^33^	1 (100)	0 (0)	0 (0)	0.8^*^	Yes (1, 100)	121
He^34^	15 (93.7)	2 (12.5)	0 (0)	<1.5	No	41 ± 5
Akay^35^	16 (100)	0 (0)	0 (0)	–	Yes (16, 100)	–
Lu^36^	50 (100)	0 (0)	0 (0)	–	No	–
Carroll^37^	1 (1000	0 (0)	0 (0)	1^*^	Yes (1, 100)	152
Zhang^38^	37 (94.8)	6 (15.3)	0 (0)	–	Yes (32, 82.1)	–
Scheer^4^	11 (100)	0 (0)	0 (0)	1.2 ± 0.5	Yes (10, 90.9)	165
Yao^39^	24 (100)	2 (8.3)	0 (0)	5.3 ± 1.4	Yes (20, 83.3)	–
Lu^21^	95 (84.8)	20 (17.8)	–	–	No	–
Burkhardt^40^	76 (97.4)	8 (10.2)	0 (0)	1.3 ± 1.1	Yes (57, 73.0)	86.2 ± 28.6
Czyz^41^	20 (95.2)	0 (0)	0 (0)	4.8 ± 1.8	Yes (19, 90.4)	111 ± 24
Czyz^42^	15 (100)	0 (0)	0 (0)	4.8 ± 1.9	No	69 ± 25
Schulder^43^	38 (97.4)	0 (0)	0 (0)	–	Yes^¶^	–
Quinn^44^	32 (96.9)	0 (0)	0 (0)	–	Yes (26, 78.7)	246
Nimsky^45^	19 (100)	0 (0)	0 (0)	4.1 ± 1.7	Yes (16, 84.2)	76.7 ± 14.8
Bernays^46^	111 (97.3)	5 (4.3)	1 (0.8)	1.9 ± 0.7	Yes (92, 80.7)	60
Kanner^47^	15 (100)	1 (6.6)	0 (0)	–	Yes^¶^	107.5
Hall^48^	38 (100)	1 (2.6)	0 (0)	–	No	–
Fontaine^49^	95 (95)	5 (5)	0 (0)	–	Yes (81, 81)	–
Bernstein^50^	10 (100)	1 (10)	0 (0)	–	Yes (9, 90)	–
Moriarty^51^	66 (97)	2 (2.9)	1 (1.4)	–	No	–
Staubert^52^	24 (92.3)	–	–	–	No	–
Rubino^53^	12 (92.3)	1 (7.6)	0 (0)	–	No	–
Hall^54^	35 (100)	1 (2.8)	0 (0)	–	Yes (24, 68.5)	–
Kollias^55^	20 (95.2)	0 (0)	0 (0)	–	Yes (20, 95.2)	–

### Diagnostic Yield, Morbidity, Mortality, Mean Lesion Size, and Procedural Time

**Figure 2.**
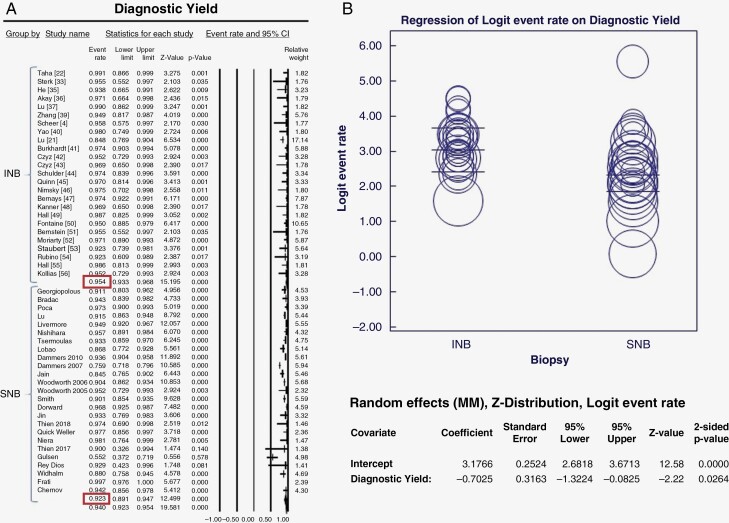
(A) Forest plot showing pooled estimates comparing the diagnostic yield of INB and SNB groups. (B) Meta-regression bubble plot for diagnostic yield with the modality of biopsy as the moderator variable.

**Figure 3.**
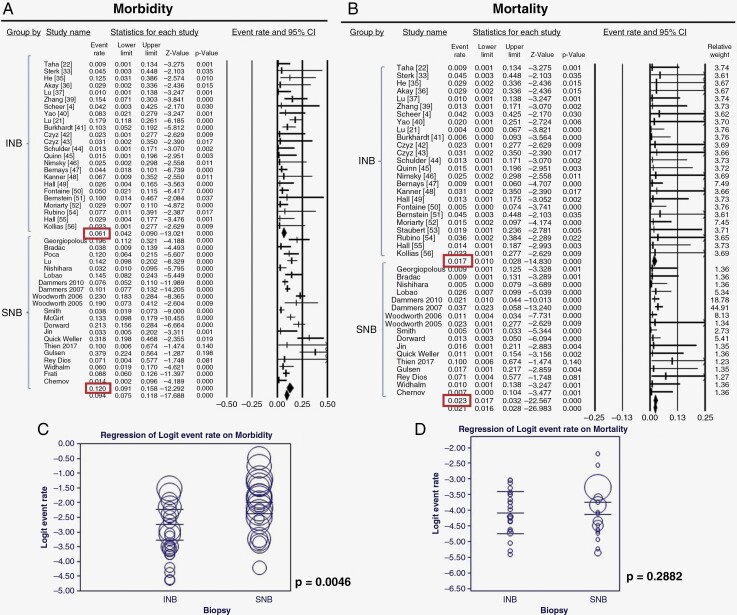
Forest plot showing pooled estimates comparing morbidity (A) and mortality (C) of INB and SNB groups. Meta-regression bubble plots for morbidity (B), and mortality (D) with modality of biopsy as the moderator variable.

**Figure 4.**
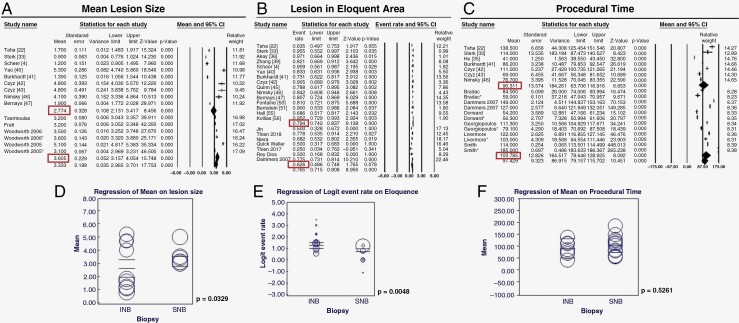
Forest plot showing pooled estimated comparing mean lesion size (A), lesion in the eloquent area (B) and procedural time (C) in INB and SNB groups. Meta-regression bubble plots for mean lesion size (D), lesion in the eloquent area (E) and procedural time (F) with the modality of biopsy as the moderator variable.

Pooled estimates for diagnostic yield for studies in the INB group were 95.4% (93.3–96.8%), in comparison to 92.3% (89.1–94.7%) in the FB/FL group, *P* = .026 (Figure 2). Data for morbidity (including the rate of postoperative hemorrhage and neurological deficit) were available in 26 studies in the INB group; and 20 studies (33 cohorts) in FB/FL group. We found the rate of morbidity to be 6.1 % (4.2–9.0%) in the INB group, which was significantly lower (*P* = .004) than the morbidity observed in the FB/FL group [12.0% (9.1–15.8%)] (Figure 3). No significant difference in mortality was found between the 2 groups: INB (1.7%) and FB/FL (2.3%), *P* = .288 (Figure 3). The mean maximal diameter of lesions in patients in the INB group (2.7 cm) was significantly smaller than the lesion size in patients in the SNB group (3.6 cm, *P* = .032) (Figure 4). The pooled estimate of procedural time for INB and SNB was comparable at 90.3 min and 103. 7 min, respectively (*P* = .526) (Figure 4).

We performed a continuous meta-regression analysis with the lesion size as the moderator variable. We do not see a significant effect of lesion size on morbidity (*P* = .724) (Supplementary Figure S1).

### Heterogeneity and Publication Bias Analysis

The Cochran’s *Q*χ^2^ and *I*^2^ tests were used to gauge the heterogeneity of results in the INB literature (Supplementary Table 1). Sensitivity and cumulative analysis were performed if *I*^2^ was >50% to determine any outlier study. The funnel plots showed no evidence of gross asymmetry for diagnostic yield, morbidity, mortality, duration of procedure, and size of lesion for the INB studies (Supplementary Figure S2), suggesting no detectable publication bias. Egger’s regression intercept test was also performed for quantitative evidence of bias (Supplementary Table 1).

## Discussion

To our knowledge, this study is the first to compare INB and SNB through a pooled meta-analysis. Our analysis indicates that, relative to SNB, INB studies more likely involved brain tumors generally deemed more difficult to biopsy or more likely to be associated with increased risk for procedural morbidity, including smaller brain tumors and tumors located in the eloquent cerebrum. Despite these differences, the pooled estimates of diagnostic yield for INB were higher than those associated with SNB (95.4% versus 92.3%, *P* = .026), and the pooled estimate of surgical morbidity for INB was half that associated with SNB (6.1% versus 12.0%, *P* = .004). The pooled estimates for procedural mortality and time were comparable between SNB and INB groups. These results support consideration for INB as a surgical technique for achieving tissue diagnosis in select brain tumors.

There are genuine advantages to real-time visualization of the biopsy trajectory relative to the target during a needle biopsy. For instance, surgical maneuvers can be adopted to adjust to technical deviations from the planned trajectory or to procedural complications, such as entry/biopsy site hematoma.^23^ These benefits highlight challenges associated with SNB, which is fundamentally a “blind” procedure that relies on mathematical triangulation of the target lesion relative to fiducials. Challenges associated with such “blind” procedures underlie the impetus for the development of INB. In this context, our meta-analysis supports the future development and adoption of INB for select lesions. Moreover, our analysis indicates that the benefits associated with INB were not associated with an overall increase in procedural time relative to SNB. Nevertheless, the benefits of INB should be weighed against the cost associated with intraoperative MRI use, the cost of the MRI-compatible stereotactic systems,^56^ as well as the learning curve associated with INB.^57^ With a pooled diagnostic yield estimate of 92.3% and procedural morbidity of 12.0%, SNB is an excellent choice for most brain tumors. For brain tumors that the surgeon considered difficult to biopsy because of size or location, or for higher-risk patients, our meta-analysis suggests consideration for INB is warranted.

As a meta-analysis, the results provided here should be considered in the context of the limitations intrinsically associated with a meta-analysis design.^58^ To address these limitations, we followed the best practiced in the published guidelines for literature review and meta-analysis.^24^ The standard metric of assessment for our meta-analysis revealed no significant heterogeneity or publication bias in the INB literature. That said, as a newer technology, the INB literature is small relative to that available for SNB. As such, our results should be interpreted in the context of potential biases associated with the limited sample size of the INB literature as well as the sample size imbalance between the INB and the SNB literature.^59^ Moreover, despite the lack of significant heterogeneity in the published INB results, the definition of eloquence and morbidity likely differ between publications, and the literature describes clinical outcomes from different MRI-compatible stereotactic systems. Irrespective of the technical nuances of these systems, the benefit of near-real-time visualization and the opportunity to adjust surgical maneuvers based on this visualization is common. Meta-analyses could either be reported as meta-analyses of proportions that focus on estimating the overall proportions in 2 treatment/modality categories, and then comparing the 2 proportions; or by meta-analyses of treatment/modality comparisons. Our reported analyses are technically chi-square test results between the 2 proportions (stereotactic needle biopsy versus intraoperative MRI-guided biopsy). There are only a few studies that reported a direct comparison between the 2 modalities and hence, performing meta-analyses with only such studies with limited overall sample size would decrease the power of the results. The formulae (*r* = 2*x* ∛ *V*.(3/4π) or *r* = √*A*/π), as used previously in the published literature, have been used to estimate approximate lesion size for similar purposes.^60^ As such, despite these limitations, we believe that our study provides a quantitative overview of the INB literature. As a study built on the current best practices of literature review and meta-analysis, we believe this study provides insights into the current INB practice as well as the associated clinical outcomes. Moreover, our study lays the foundation for future studies and technology development.

The important causes for reported peri-procedural morbidity and mortality were an expanding intraparenchymal hematoma or worsening edema postbiopsy for which the patients were taken back to the operating room. In this context, one must carefully plan the biopsy trajectory to avoid entrance through the sulci, or any blood vessel. It is also important to monitor neurological exams in the postoperative period for any clinical signs of worsening edema. One reported mortality occurred before the biopsy was performed, hence, securing a preoperative clearance from our anesthesia colleagues cannot be underestimated.

Although the cost-effectiveness of intraoperative MRI for the treatment of high-grade gliomas has been well established in the literature,^56^ but the same still needs to be established for the purposes of biopsy. One can argue that on one hand with intraoperative MRI the higher diagnostic yield, lower postoperative complications and the ability to correct the trajectory in real time for brain shifts make this modality cost-effective, while on the other hand, the cost of MRI with relatively longer anesthesia duration and cumulative operating room time adds up to the total cost of the procedure. Future studies should focus toward analyzing the cost of intraoperative MRI-guided biopsies versus conventional stereotactic needle biopsies.

## Conclusion

Our meta-analysis indicates that the INB literature more likely involves smaller brain tumors or tumors located in the eloquent cerebrum. Despite these differences, INB is associated with higher diagnostic yield and lower procedural morbidity.

## Supplementary Material

vdad129_suppl_Supplementary_Figures_S1

vdad129_suppl_Supplementary_Tables_S1

vdad129_suppl_Supplementary_Figures_S2

vdad129_suppl_Supplementary_Data

## References

[1] Cordova JS , Gurbani SS, Olson JJ, et al. A systematic pipeline for the objective comparison of whole-brain spectroscopic MRI with histology in biopsy specimens from grade III glioma. Tomography. 2016;2(2):106–116. 27489883 10.18383/j.tom.2016.00136 PMC4968944

[2] Akshulakov SK , Kerimbayev TT, Biryuchkov MY, et al. Current trends for improving safety of stereotactic brain biopsies: Advanced optical methods for vessel avoidance and tumor detection. Front Oncol. 2019;9(9):947. 31632903 10.3389/fonc.2019.00947 PMC6783564

[3] Jain D , Sharma MC, Sarkar C, et al. Correlation of diagnostic yield of stereotactic brain biopsy with number of biopsy bits and site of the lesion. Brain Tumor Pathol. 2006;23:71–75. 18095122 10.1007/s10014-006-0204-y

[4] Scheer JK , Hamelin T, Chang L, et al. Real-time magnetic resonance imaging-guided biopsy using smartframe^®^ stereotaxis in the setting of a conventional diagnostic magnetic resonance imaging suite. Oper Neurosurg (Hagerstown). 2017;13(3):329–337. 28521346 10.1093/ons/opw035

[5] Kellermann SG , Hamisch CA, Rueß D, et al. Stereotactic biopsy in elderly patients: risk assessment and impact on treatment decision. J Neurooncol. 2017;134(2):303–307. 28639133 10.1007/s11060-017-2522-9

[6] Chabaane M , Amelot A, Riche M, et al. Efficacy of a second brain biopsy for intracranial lesions after initial negativity. J Clin Neurol. 2020;16(4):659–667. 33029973 10.3988/jcn.2020.16.4.659 PMC7542000

[7] Dammers R , Haitsma IK, Schouten JW, et al. Safety and efficacy of frameless and frame-based intracranial biopsy techniques. Acta Neurochir (Wien). 2008;150:23–29. 18172567 10.1007/s00701-007-1473-x

[8] Dammers R , Schouten JW, Haitsma IK, et al. Towards improving the safety and diagnostic yield of stereotactic biopsy in a single centre. Acta Neurochir (Wien). 2010;152:1915–1921. 20680649 10.1007/s00701-010-0752-0 PMC2956059

[9] Dhawan S , Venteicher AS, Butler WE, Carter BS, Chen CC. Clinical outcomes as a function of the number of samples taken during stereotactic needle biopsies: A meta-analysis. J Neurooncol. 2021;154:1–11. 34251602 10.1007/s11060-021-03785-9

[10] McGirt MJ , Woodworth GF, Coon AL, et al. Independent predictors of morbidity after image-guided stereotactic brain biopsy: a risk assessment of 270 cases. J Neurosurg. 2005;102:897–901. 15926716 10.3171/jns.2005.102.5.0897

[11] Woodworth GF , McGirt MJ, Samdani A, et al. Frameless image-guided stereotactic brain biopsy procedure: diagnostic yield, surgical morbidity, and comparison with the frame-based technique. J Neurosurg. 2006;104:233–237. 10.3171/jns.2006.104.2.233 16509497

[12] Waters JD , Gonda DD, Reddy H, et al. Diagnostic yield of stereotactic needle-biopsies of sub-cubic centimeter intracranial lesions. Surg Neurol Int. 2013;4(Suppl. 3):S176–S181. 23682345 10.4103/2152-7806.110677 PMC3654772

[13] Dhawan S , He Y, Bartek J, Jr, Alattar AA, Chen CC. Comparison of frame-based versus frameless intracranial stereotactic biopsy: Systematic review and meta-analysis. World Neurosurg. 2019;127:607–616.e4. 30974279 10.1016/j.wneu.2019.04.016

[14] Patel KS , Carter BS, Chen CC. Role of biopsies in the management of intracranial gliomas. Prog Neurol Surg. 2018;30:232–243. 29241178 10.1159/000464439

[15] Barnett GH , Miller DW, Weisenberger J. Frameless stereotaxy with scalp-applied fiducial markers for brain biopsy procedures: Experience in 218 cases. J Neurosurg. 1999;91(4):569–576. 10507376 10.3171/jns.1999.91.4.0569

[16] Fitzpatrick JM. The role of registration in accurate surgical guidance. Proc Inst Mech Eng H. 2010;224(5):607–622. 20718266 10.1243/09544119JEIM589 PMC4860017

[17] Mitsui T , Fujii M, Tsuzaka M, et al. Skin shift and its effect on navigation accuracy in image-guided neurosurgery. Radiol Phys Technol. 2011;4(1):37–42. 20830539 10.1007/s12194-010-0103-0

[18] Zrinzo L. Pitfalls in precision stereotactic surgery. Surg Neurol Int. 2012;3(Suppl. 1):S53–S61. 22826812 10.4103/2152-7806.91612 PMC3400482

[19] Winkler D , Tittgemeyer M, Schwarz J, et al. The first evaluation of brain shift during functional neurosurgery by deformation field analysis. J Neurol Neurosurg Psychiatry. 2005;76:1161–1163. 16024899 10.1136/jnnp.2004.047373 PMC1739770

[20] Ivan ME , Yarlagadda J, Saxena AP, et al. Brain shift during bur hole-based procedures using interventional MRI. J Neurosurg. 2014;121(1):149–160. 24785326 10.3171/2014.3.JNS121312

[21] Lu Y , Yeung C, Radmanesh A, et al. Comparative effectiveness of frame-based, frameless, and intraoperative magnetic resonance imaging-guided brain biopsy techniques. World Neurosurg. 2015;83(3):261–268. 25088233 10.1016/j.wneu.2014.07.043 PMC4450019

[22] Taha BR , Osswald CR, Rabon M, et al. Learning curve associated with clear point neuro navigation system: A case series. World Neurosurg X. 2021;13:100115. 35028557 10.1016/j.wnsx.2021.100115 PMC8739880

[23] Li H , Zheng C, Rao W, et al. The risk factors of hemorrhage in stereotactic needle biopsy for brain lesions in a large cohort: 10 years of experience in a single center. Chin Neurosurg J. 2022;8(1):40. 36494749 10.1186/s41016-022-00307-y PMC9732999

[24] Page MJ , McKenzie JE, Bossuyt PM, et al. The PRISMA 2020 statement: An updated guideline for reporting systematic reviews. BMJ. 2021;372:n71. 33782057 10.1136/bmj.n71 PMC8005924

[25] Dhawan S , Chen CC. 2023. Available at http://www.crd.york.ac.uk/PROSPERO/display_record.asp?ID=CRD42020152735. Date accessed May 15, 2023.

[26] Lo CK , Mertz, D, Loeb M. Newcastle–Ottawa scale: Comparing reviewers’ to authors’ assessments. BMC Med Res Methodol. 2014;14:45. 10.1186/1471-2288-14-45 PMC4021422 24690082

[27] DerSimonian R , Laird N. Meta-analysis in clinical trials. Control Clin Trials. 1986;7(3):177–188. 3802833 10.1016/0197-2456(86)90046-2

[28] Higgins JP , Thompson SG, Deeks JJ, Altman DG. Measuring inconsistency in meta-analyses. BMJ. 2003;327(7414):557–560. 12958120 10.1136/bmj.327.7414.557 PMC192859

[29] Duval S , Tweedie R. Trim and fill: A simple funnel-plot-based method of testing and adjusting for publication bias in meta-analysis. Biometrics. 2000;56(2):455–463. 10877304 10.1111/j.0006-341x.2000.00455.x

[30] Egger M , Davey Smith G, Schneider M, Minder C. Bias in meta-analysis detected by a simple, graphical test. BMJ. 1997;315(7109):629–634. 9310563 10.1136/bmj.315.7109.629 PMC2127453

[31] Mathur MB , VanderWeele TJ. Sensitivity analysis for publication bias in meta-analyses. J R Stat Soc Ser C Appl Stat. 2020;69(5):1091–1119. 10.1111/rssc.12440 PMC7590147 33132447

[32] Sterk B , Taha B, Osswald C, et al. Initial clinical experience with clearpoint smartframe array-aided stereotactic procedures. World Neurosurg. 2022;162:e120–e130. 35231619 10.1016/j.wneu.2022.02.095

[33] Bartek J, Jr, Alattar A, Jensdottir M, Chen CC. Biopsy and ablation of H3K27 glioma using skull-mounted smartframe device: Technical case report. World Neurosurg. 2019;127:436–441. 30974271 10.1016/j.wneu.2019.04.029

[34] He X , Liu M, Liu C, et al. Real-time MR-guided brain biopsy using 10-T open MRI scanner. Eur Radiol. 2019;29(1):85–92. 29948073 10.1007/s00330-018-5531-y

[35] Akay A , Işlekel S. MRI-guided frame-based stereotactic brainstem biopsy procedure: A single-center experience. Neurocirugia (English Edition). 2019;30(4):167–172. 31000332 10.1016/j.neucir.2019.03.001

[36] Lu CY , Xu ZS, Ye X. Evaluation of intraoperative MRI-assisted stereotactic brain tissue biopsy: A single-center experience in China. Chin Neurosurg J. 2019;5:4. 32922904 10.1186/s41016-019-0152-0 PMC7398305

[37] Carroll KT , Lochte BC, Chen JY, et al. Intraoperative magnetic resonance imaging-guided biopsy in the diagnosis of suprasellar langerhans cell histiocytosis. World Neurosurg. 2018;112:6–13. 29317365 10.1016/j.wneu.2017.12.184

[38] Zhang JS , Qu L, Wang Q, et al. Intraoperative visualisation of functional structures facilitates safe frameless stereotactic biopsy in the motor eloquent regions of the brain. Br J Neurosurg. 2018;32(4):372–380. 29260585 10.1080/02688697.2017.1416059

[39] Yao C , Lv S, Chen H, et al. The clinical utility of multimodal MR image-guided needle biopsy in cerebral gliomas. Int J Neurosci. 2016;126(1):53–61. 25539452 10.3109/00207454.2014.992429

[40] Burkhardt JK , Neidert MC, Woernle CM, Bozinov O, Bernays R-L. Intraoperative low-field MR-guided frameless stereotactic biopsy for intracerebral lesions. Acta Neurochir (Wien). 2013;155(4):721–726. 23435865 10.1007/s00701-013-1639-7

[41] Czyż M , Tabakow P, Weiser A, et al. The safety and effectiveness of low field intraoperative MRI guidance in frameless stereotactic biopsies of brain tumours-design and interim analysis of a prospective randomized trial. Neurosurg Rev. 2014;37(1):127–137. 23821131 10.1007/s10143-013-0486-6 PMC3889652

[42] Czyż M , Tabakow P, Jarmundowicz W, Lechowicz-Głogowska B. Intraoperative magnetic resonance-guided frameless stereotactic biopsies – initial clinical experience. Neurol Neurochir Pol. 2012;46(2):157–160. 10.5114/ninp.2012.28258 22581597

[43] Schulder M , Spiro D. Intraoperative MRI for stereotactic biopsy. Acta Neurochir Suppl. 2011;109:81–87. 20960325 10.1007/978-3-211-99651-5_13

[44] Quinn J , Spiro D, Schulder M. Stereotactic brain biopsy with a low-field intraoperative magnetic resonance imager. Neurosurgery. 2011;68(1 Suppl. Operative):217–24; discussion 224. 21206306 10.1227/NEU.0b013e31820826c2

[45] Nimsky C , Fujita A, Ganslandt O, et al. Frameless stereotactic surgery using intraoperative high-field magnetic resonance imaging. Neurol Med Chir (Tokyo). 2004;44(10):522–33; discussion 534. 15633465 10.2176/nmc.44.522

[46] Bernays RL , Kollias SS, Khan N, et al. Histological yield, complications, and technological considerations in 114 consecutive frameless stereotactic biopsy procedures aided by open intraoperative magnetic resonance imaging. J Neurosurg. 2002;97(2):354–362. 12186464 10.3171/jns.2002.97.2.0354

[47] Kanner AA , Vogelbaum MA, Mayberg MR, Weisenberger JP, Barnett GH. Intracranial navigation by using low-field intraoperative magnetic resonance imaging: preliminary experience. J Neurosurg. 2002;97(5):1115–1124. 12450034 10.3171/jns.2002.97.5.1115

[48] Hall WA , Liu H, Martin AJ, Maxwell RE, Truwit CL. Brain biopsy sampling by using prospective stereotaxis and a trajectory guide. J Neurosurg. 2001;94(1):67–71. 11147900 10.3171/jns.2001.94.1.0067

[49] Fontaine D , Dormont D, Hasboun D, et al. Magnetic resonance-guided stereotactic biopsies: results in 100 consecutive cases. Acta Neurochir (Wien). 2000;142(3):249–55; discussion 255. 10819254 10.1007/s007010050032

[50] Bernstein M , Al-Anazi AR, Kucharczyk W, et al. Brain tumor surgery with the Toronto open magnetic resonance imaging system: preliminary results for 36 patients and analysis of advantages, disadvantages, and future prospects. Neurosurgery. 2000;46(4):900–7; discussion 907. 10764263 10.1097/00006123-200004000-00023

[51] Moriarty TM , Quinones-Hinojosa A, Larson PS, et al. Frameless stereotactic neurosurgery using intraoperative magnetic resonance imaging: Stereotactic brain biopsy. Neurosurgery. 2000;47(5):1138–45; discussion 1145. 11063107 10.1097/00006123-200011000-00023

[52] Staubert A , Vester M, Tronnier VM, et al. Interventional MRI-guided brain biopsies using inductively coupled surface coils. Magn Reson Med. 2000;43(2):278–283. 10680692 10.1002/(sici)1522-2594(200002)43:2<278::aid-mrm15>3.0.co;2-e

[53] Rubino GJ , Farahani K, McGill D, et al. Magnetic resonance imaging-guided neurosurgery in the magnetic fringe fields: The next step in neuronavigation. Neurosurgery. 2000;46(3):643–53; discussion 653. 10719861 10.1097/00006123-200003000-00023

[54] Hall WA , Martin AJ, Liu H, et al. Brain biopsy using high-field strength interventional magnetic resonance imaging. Neurosurgery. 1999;44(4):807–13; discussion 813. 10201306 10.1097/00006123-199904000-00067

[55] Kollias SS , Bernays R, Marugg RA, et al. Target definition and trajectory optimization for interactive MR-guided biopsies of brain tumors in an open configuration MRI system. J Magn Reson Imaging. 1998;8(1):143–159. 9500274 10.1002/jmri.1880080127

[56] Abraham P , Sarkar R, Brandel MG, et al. Cost-effectiveness of intraoperative MRI for treatment of high-grade gliomas. Radiology. 2019;291(3):689–697. 30912721 10.1148/radiol.2019182095 PMC6543900

[57] Kesserwan MA , Shakil H, Lannon M, et al. Frame-based versus frameless stereotactic brain biopsies: A systematic review and meta-analysis. Surg Neurol Int. 2021;12:52. 33654555 10.25259/SNI_824_2020 PMC7911151

[58] Lyman GH , Kuderer NM. The strengths and limitations of meta-analyses based on aggregate data. BMC Med Res Methodol. 2005;5:14. 15850485 10.1186/1471-2288-5-14 PMC1097735

[59] Lin L. Bias caused by sampling error in meta-analysis with small sample sizes. PLoS One. 2018;13(9):e0204056. 30212588 10.1371/journal.pone.0204056 PMC6136825

[60] Routman DM , Bian SX, Diao K, et al. The growing importance of lesion volume as a prognostic factor in patients with multiple brain metastases treated with stereotactic radiosurgery. Cancer Med. 2018;7(3):757–764. 29441722 10.1002/cam4.1352 PMC5852368

