# Supplementary figures and images for: Comparison meta-analysis of intraoperative MRI-guided needle biopsy versus conventional stereotactic needle biopsies
Source: Neurooncol Adv. 2023 Oct 10;6(1):vdad129. doi: 10.1093/noajnl/vdad129 (PMC10771274; doi:10.1093/noajnl/vdad129)

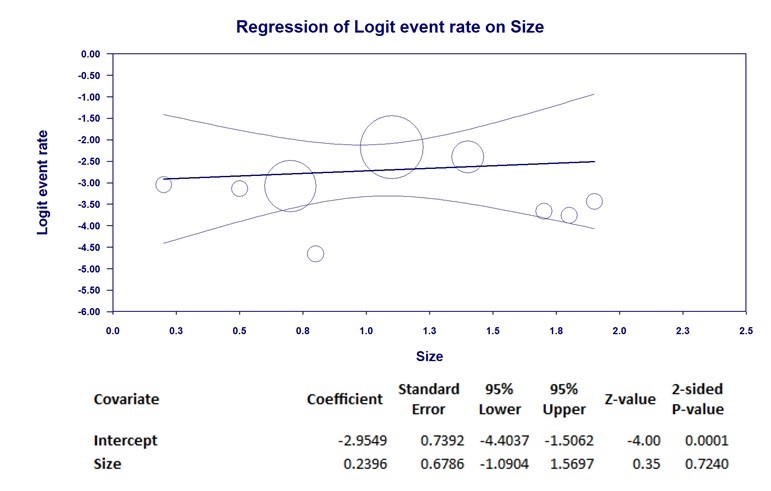

Supplement: vdad129_suppl_Supplementary_Figures_S1 [file vdad129_suppl_supplementary_figures_s1.jpeg]

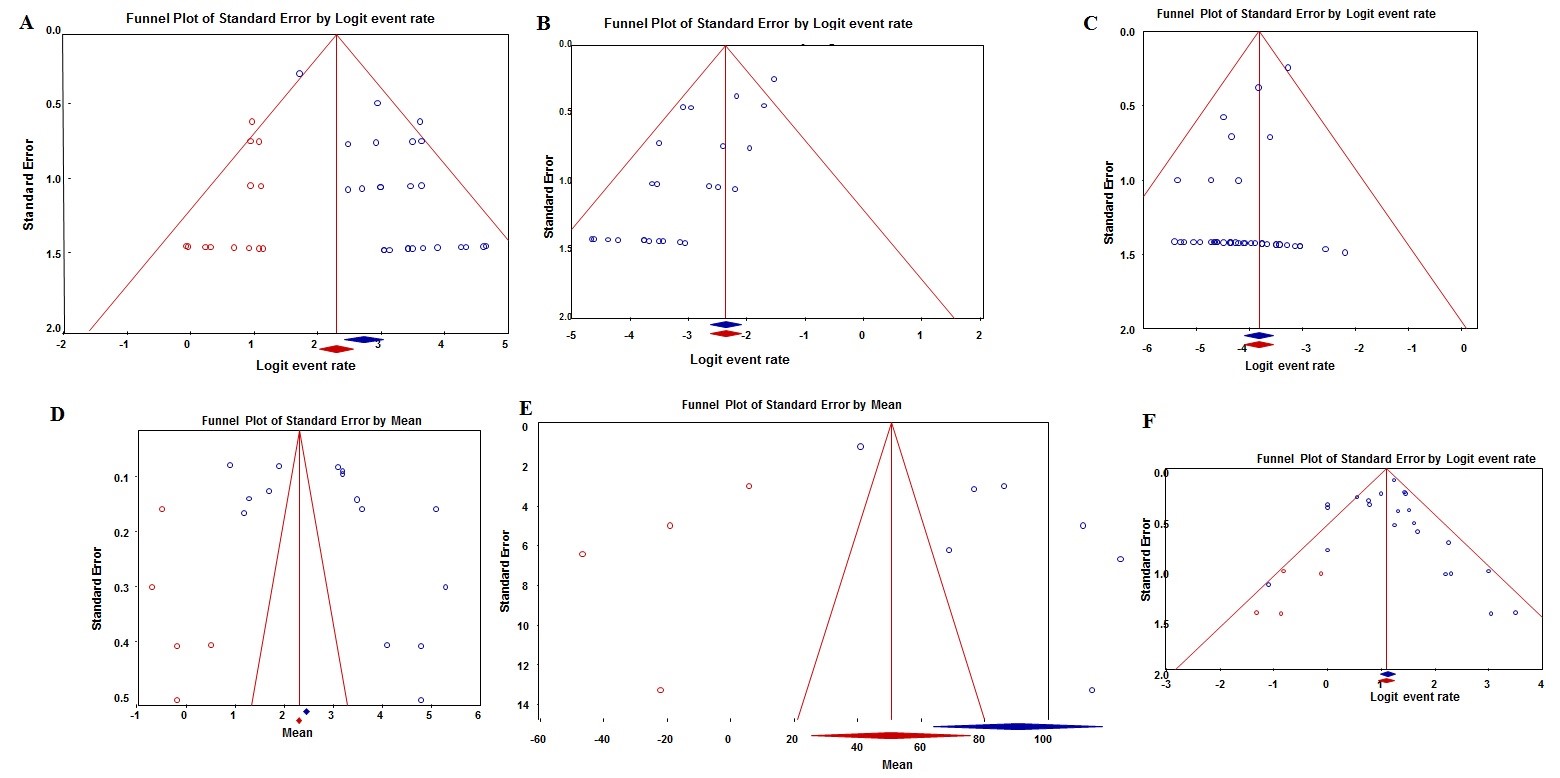

Supplement: vdad129_suppl_Supplementary_Figures_S2 [file vdad129_suppl_supplementary_figures_s2.jpeg]
