# Supplementary material for: Comparison meta-analysis of intraoperative MRI-guided needle biopsy versus conventional stereotactic needle biopsies
Source: Neurooncol Adv. 2023 Oct 10;6(1):vdad129. doi: 10.1093/noajnl/vdad129 (PMC10771274; doi:10.1093/noajnl/vdad129)
Supplement: vdad129_suppl_Supplementary_Tables_S1 [file vdad129_suppl_supplementary_tables_s1.docx]

**Supplementary Table S1**: Heterogeneity analysis and Egger regression intercept for various parameters, and results of sensitivity and cumulative analysis for detection of outlier study.

| **Parameter(s)** | **I^2^** | **Egger Regression Intercept** | | **Outlier study on sensitivity/cumulative analysis** | | | | | |
| --- | --- | --- | --- | --- | --- | --- | --- | --- | --- |
|  |  | **Intercept** | **p value** |  |  |  |  |  |  |
| Size of lesion | 98.3 | 8.91 | 0.15 | No | | | | | |
| Procedural time | 98.9 | 12.31 | 0.01 | No | | | | | |
| Eloquent location | 34.9 | 0.25 | 0.65 | No | | | | | |
| Diagnostic Yield | 19.1 | 1.6 | 0.01 | No | | | | | |
| Morbidity | 37.8 | -1.71 | 0.01 | No | | | | | |
| Mortality | 0 0.00 | -0.56 | 0.01 | No | | | | | |
