# Supplementary material for: Comparison meta-analysis of intraoperative MRI-guided needle biopsy versus conventional stereotactic needle biopsies
Source: Neurooncol Adv. 2023 Oct 10;6(1):vdad129. doi: 10.1093/noajnl/vdad129 (PMC10771274; doi:10.1093/noajnl/vdad129)
Supplement: vdad129_suppl_Supplementary_Data [file vdad129_suppl_supplementary_data.docx]

**Supplementary Figure S1:** Bubble plot showing continuous meta-regression analysis of morbidity with lesion size as the moderator variable (p=0.724).

**Supplementary Figure S2:** Funnel plots for publication bias assessment for A) Diagnostic Yield, B) Morbidity, C) Mortality, D) Mean lesion size, E) Procedural Time, and F) location of lesion in eloquent brain. Blue circles indicate observed studies; red circles, imputed studies; blue diamond, plot for observed studies; red diamond, plot for both observed and imputed studies from trim and fill analysis. There is no gross asymmetry observed in funnel plots suggestive of absence of publication bias.

**Supplementary Table S1**: Heterogeneity analysis and Egger regression intercept for various parameters, and results of sensitivity and cumulative analysis for detection of outlier study.
